# Rapid Host Defense against *Aspergillus fumigatus* Involves Alveolar Macrophages with a Predominance of Alternatively Activated Phenotype

**DOI:** 10.1371/journal.pone.0015943

**Published:** 2011-01-05

**Authors:** Shikha Bhatia, Mingjian Fei, Manohar Yarlagadda, Zengbiao Qi, Shizuo Akira, Shinobu Saijo, Yoichiro Iwakura, Nico van Rooijen, Gregory A. Gibson, Claudette M. St. Croix, Anuradha Ray, Prabir Ray

**Affiliations:** 1 Division of Pulmonary, Allergy and Critical Care Medicine, Department of Medicine, University of Pittsburgh School of Medicine, Pittsburgh, Pennsylvania, United States of America; 2 Department of Immunology, University of Pittsburgh School of Medicine, Pittsburgh, Pennsylvania, United States of America; 3 Laboratory of Host Defense, WPI Immunology Frontier Research Center, Osaka, Japan; 4 Center for Experimental Medicine and Systems Biology, Institute of Medical Science, University of Tokyo, Tokyo, Japan; 5 CREST, Japan Science and Technology Agency, Saitama, Japan; 6 Department of Molecular Cell Biology, Vrije Universiteit, Vanderbilt University Medical Center, Amsterdam, The Netherlands; 7 Department of Cell Biology, University of Pittsburgh School of Medicine, Pittsburgh, Pennsylvania, United States of America; 8 Department of Environmental and Occupational Health, University of Pittsburgh School of Medicine, Pittsburgh, Pennsylvania, United States of America; University of California Los Angeles, United States of America

## Abstract

The ubiquitous fungus *Aspergillus fumigatus* is associated with chronic diseases such as invasive pulmonary aspergillosis in immunosuppressed patients and allergic bronchopulmonary aspergillosis (ABPA) in patients with cystic fibrosis or severe asthma. Because of constant exposure to this fungus, it is critical for the host to exercise an immediate and decisive immune response to clear fungal spores to ward off disease. In this study, we observed that rapidly after infection by *A. fumigatus*, alveolar macrophages predominantly express Arginase 1 (Arg1), a key marker of alternatively activated macrophages (AAMs). The macrophages were also found to express Ym1 and CD206 that are also expressed by AAMs but not NOS2, which is expressed by classically activated macrophages. The expression of Arg1 was reduced in the absence of the known signaling axis, IL-4Rα/STAT6, for AAM development. While both Dectin-1 and TLR expressed on the cell surface have been shown to sense *A. fumigatus*, fungus-induced Arg1 expression in CD11c^+^ alveolar macrophages was not dependent on either Dectin-1 or the adaptor MyD88 that mediates intracellular signaling by most TLRs. Alveolar macrophages from WT mice efficiently phagocytosed fungal conidia, but those from mice deficient in Dectin-1 showed impaired fungal uptake. Depletion of macrophages with clodronate-filled liposomes increased fungal burden in infected mice. Collectively, our studies suggest that alveolar macrophages, which predominantly acquire an AAM phenotype following *A. fumigatus* infection, have a protective role in defense against this fungus.

## Introduction

*Aspergillus fumigatus* is a ubiquitous fungus that is efficiently cleared by immunocompetent hosts. Inability to efficiently clear Aspergillus under conditions of immune suppression, which is a common occurrence in organ transplant patients, induces severe invasive disease [1]. In patients with cystic fibrosis or severe asthma fungal clearance is also impaired which causes allergic bronchopulmonary aspergillosis (ABPA) [1], [2]. In the lung, macrophages and neutrophils are the key cell types involved in defense against various pathogens including Aspergillus [3]. Macrophages constitute an important and primary line of defense against any infection. These cells not only serve a role in pathogen phagocytosis but they can also function as modulators of the immune response [4]. Development, behavior and functional properties of macrophages are influenced by various environmental cues to which these cells are exposed [5], [6], [7]. Several phenotypes or classifications of macrophages have been described. However, they can be best divided into two broad categories. Classically Activated Macrophages (CAMs) induced by IFN-γ are designated as M1 macrophages [7], [8]. Alternatively Activated Macrophages (AAMs) or M2 macrophages are so designated because of the ability of IL-4 to enhance expression of mannose receptor, considered a distinctive feature of these macrophages [9]. While the M1/M2 designation is still used in the literature, the M2 subclass has expanded to include macrophages with diverse phenotypes and functions [7], [8], [10].

The most important function of CAMs is engulfment and destruction of microbial agents. Activated CAMs produce pro-inflammatory cytokines such as TNFα and IL-6 and also show marked upregulation of nitric oxide synthase (NOS2) associated with NO production that together help in the destruction of the phagocytosed pathogens [7], [8], [10]. AAMs have been best studied in the context of infections by helminths [7], [8]. However, AAMs have been also noticed during infections by intracellular bacteria [11]or viruses [12], [13] and in other disease conditions such as allergic airways disease in mice [14], [15], diabetes [16], [17] and cancer [18], [19]. Various markers have been identified for AAMs like Arginase1 (Arg1), Chi3l3(Ym1), Chi3l4(Ym2), Fizz1(Found in Inflammatory Zone1) and macrophage mannose receptor (CD206). However, thus far Arg1 is regarded as the prototype activation marker for AAMs in murine macrophages [7]. Arg1 expressed by AAMs metabolizes L-Arginine (L-Arg), the common substrate for both NOS2 and Arg1, to produce orninthine and urea. Arg1 activation generates polyamines and hydroxyprolines that help in repair processes after tissue injury caused by parasitic infections and suppress Th2 effector functions [20], [21].

Recently, the function of AAMs was addressed either by depleting them or by using mouse models deficient in their signature molecules like Arg1 and Fizz1. Thus, in infections by *Nippostrongylus brasiliensis* or *Schistosoma mansoni*, Arg1- and Fizz1-expressing AAMs were shown to be suppressors of Th2 inflammation in the lung [21], [22], [23]. Furthermore, ablation of Arg1 specifically in macrophages and neutrophils exacerbated schistosomiasis and the presence of this enzyme was necessary for downregulating chronic inflammation and suppressing fibrosis [21]. In contrast, during infections by intracellular pathogens such as *Toxoplasma gondii* and *Mycobacterium bovis*, CAMs were found to upregulate expression of Arg1 with concomitant suppression of NO production due to competition for the common substrate L-Arg [11]. This process interfered with microbial killing since mice lacking Arg1 showed higher survival rate [11]. CD 4 T cell memory response that helped clear *Heligmosomoides polygyrus* was found to be facilitated by AAMs [24].

In the present study, we explored the nature of the early innate immune response to *Aspergillus fumigatus* infection of the lung. We show that after fungal infection, AAMs expressing Arg1, Ym1 and CD206 develop in the lung as early as 6 hours after infection. The expression of Arg1 in BAL CD11c^+^ cells was only partially dependent on IL-4Rα/STAT6. Moreover, Arg1 expression was also not dependent on Dectin-1 or MyD88, pathways associated with fungal recognition and induction of immune responses [25], [26], [27], [28], [29], [30], [31], [32], [33]. However, Dectin-1 was important for the phagocytosis of Aspergillus conidia. Depletion of macrophages by clodronate-filled liposomes delayed the clearance of fungus after infection even though neutrophil numbers increased upon clodronate treatment. Alveolar macrophages from WT mice efficiently phagocytosed fungal conidia, but those from mice deficient in Dectin-1 showed impaired fungal uptake. Since Arg1, constitutively expressed by neutrophils, was previously associated with antifungal activity [34], switching on expression of this enzyme in alveolar macrophages highlights an important antifungal defense mechanism. Taken together, our data suggest that rapid induction of Arg1 in alveolar macrophages after *A. fumigatus* infection is a key antifungal defense mechanism employed by the infected host to eliminate the fungus.

## Results

### *A. fumigatus* infection induces the prototypic marker of Alternatively Activated Macrophages (AAMs) Arginase 1 in the lung

**Figure 1:**
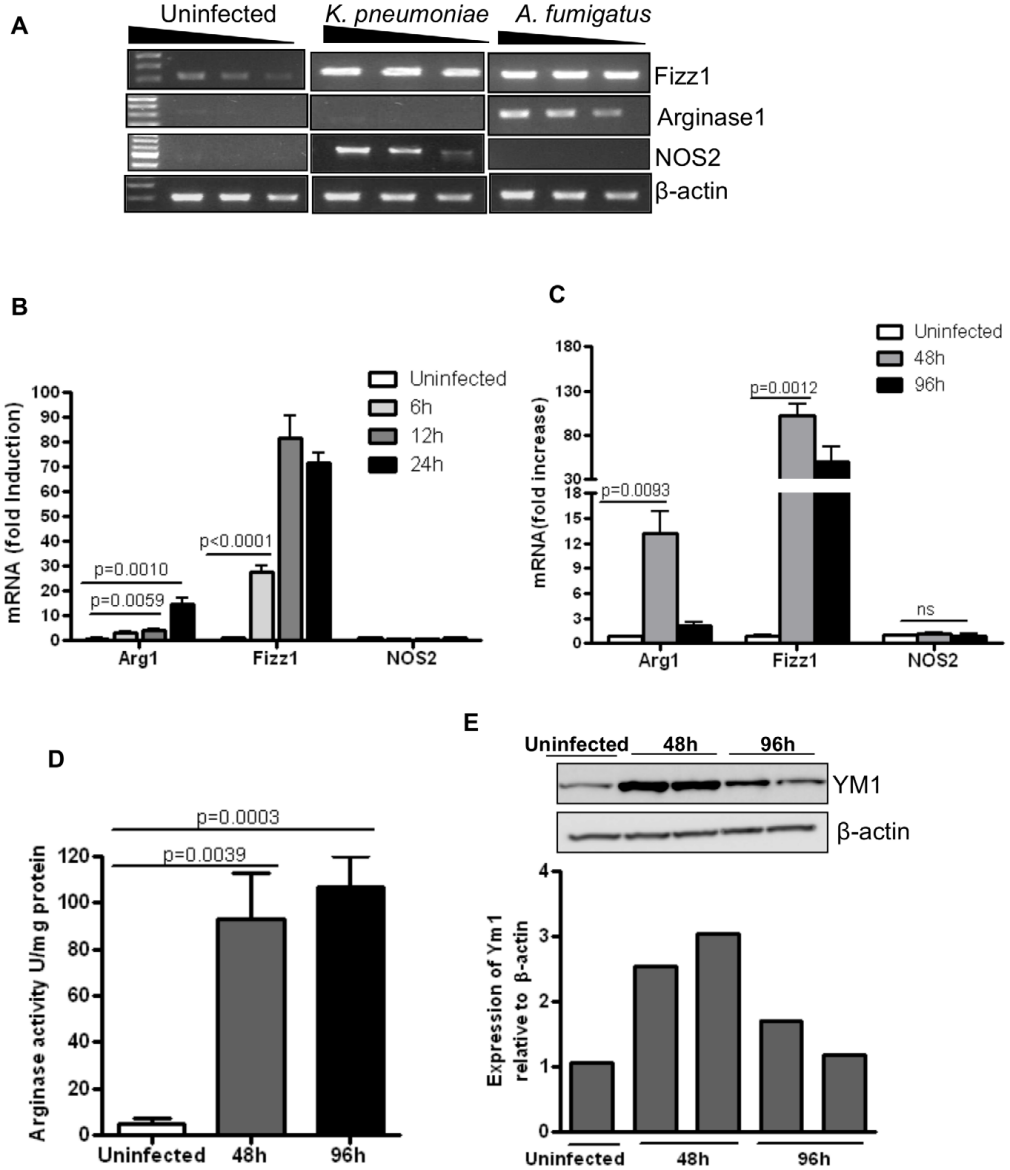
Infection by *Aspergillus fumigatus* induces markers of Alternatively Activated Macrophages in the lung. (A) Mice were infected with 100 cfu of *K. pneumoniae* or 50×10^6^ resting conidia (RC) of *A. fumigatus* given intratracheally and lungs were harvested after 4 days (Klebsiella) or 48 hours (Aspergillus) of infection for total RNA extraction. RT-PCR was performed to measure mRNA expression of Arg1, Fizz1 and NOS2. The results shown were generated using RNA from 1 mouse (n = 4) with the PCR products in the different lanes generated with increasing dilutions of cDNA. β-actin expression was used as an internal control. mRNA expression corresponding to the various genes in infected lungs was compared with that expressed in the lungs of uninfected mice. Mice were infected with 50×10^6^ RC or given PBS intratracheally (uninfected group). The expression of Arg1, NOS2 and Fizz1 was analyzed by quantitative RT-PCR (B) at 6, 12 and 24 hours (C) at 48 and 96 hours p. i. The fold increase in expression for each gene is expressed relative to that in uninfected mice using Gus-β expression for normalization. Values shown are mean ± SEM. (D) Arginase activity expressed as U/mg protein was measured in protein extracts made from lungs 48 and 96 hours p. i. (E) Immunoblotting of YM1 was performed using protein extracts made from lungs using anti-YM1 antibody. β-actin expression was examined as loading control and the intensity of YM1 band was quantified relative to that of β-actin. Data shown are representatives of two independent experiments (n = 4–6 mice in each group).

We first compared the innate immune response in the lung to two very different pathogens, the extracellular bacterium *Klebsiella pneumoniae*, and the fungus *Aspergillus fumigatus*. Mice were either left uninfected or infected intratracheally with 100 cfu (colony forming units) of *K. pneumoniae* or 50×10^6^ resting conidia (RC) of *A. fumigatus*. Lungs were harvested after 4 days of infection with *K. pneumoniae* or 48 hours of infection with *A. fumigatus* and mRNA expression for various AAM markers was determined by semi-quantitative RT-PCR techniques. The expression of Fizz1/Relm-α, a protein expressed by AAMs, epithelial cells and eosinophils was increased in the lung in both the infection models (Figure 1A). Whereas *K. pneumoniae* infection promoted NOS2 gene expression, infection by *A. fumigatus* caused increased Arg1 expression in the lung (Figure 1A). Since CAMs express NOS2 while AAMs express Arg1, these results suggested that *A. fumigatus* infection induces AAM-type cells. We next infected mice with different numbers of RC (2.5–50×10^6^ per mouse) of *A. fumigatus* and harvested the lungs at 48 hours post-infection (p.i.). As shown in Figure S1A, the expression of genes such as Arg1, Fizz1 and Ym1 increased with increasing doses of RC while that of NOS2 did not increase much over that detected in control PBS-treated mice. Arg1, Fizz1 and Ym1 are genes associated with AAMs while NOS2 is expressed by CAMs. Further, using the dose of 50×10^6^ RC for infection, we harvested lungs at different times after infection to assess expression of AAM-associated genes (Figure S1B). Of note, at 48 hours after infection, a low level of Arg1 was noted (data not shown) in the lungs of Klebsiella-infected mice which disappeared after 4 days (as shown). While Arg1 was upregulated, NOS2 expression was not detected at any time point (from 24–120 hours) in the lungs of Aspergillus-infected mice (Figure S1B). Collectively, the results showed peak expression of AAM-associated genes in the lung at 48 hours p.i.

We next infected mice with 50×10^6^ RC to assess expression of AAM-associated molecules at both mRNA and protein levels at time points earlier than 48 hours p.i. to determine whether the expression of Arg1 but not NOS2 was evident from times very early after infection. Whole lung tissue was isolated from infected mice at 6, 12, 24, 48 and 96 hours p.i. and processed for RNA. As early as 6 hours after infection, the expression of Arg1 and Fizz1 was detected in the infected lungs when compared to uninfected controls but no induction of NOS2 was noticed (Figure 1B). The steady state levels of both Arg1 and Fizz1 mRNA peaked at 48 hours p.i. The expression of Arg1 and Fizz1 was 13- and 90-fold higher in the infected lungs compared to that in uninfected controls (Figure 1C). Further, the expression of Arg1 decreased substantially at 96 hours p.i. while that of Fizz1 was reduced but remained elevated (Figure 1C). Comparatively, the expression of NOS2, the signature marker for CAMs, did not appreciably increase in the infected lungs at any of these time points (Figure 1B,C and Figure S1B).

In addition to investigating the expression of AAM-associated genes at the mRNA level, we also examined expression of the corresponding proteins. Arg1 enzyme activity and expression of YM1 protein were assessed. Arg1 enzyme activity was high at both 48 and 96 hours p.i. (Figure 1D) even though decreased Arg1 mRNA level was noted at the later time point (Figure 1C). Increased YM1 protein expression was noted at 48 hours p.i. which decreased at 96 hours after infection (Figure 1E). Beyond 96 hours, expression of both proteins declined (data not shown).

### Characterization of bronchoalveolar lavage cells after *A. fumigatus* infection

**Figure 2:**
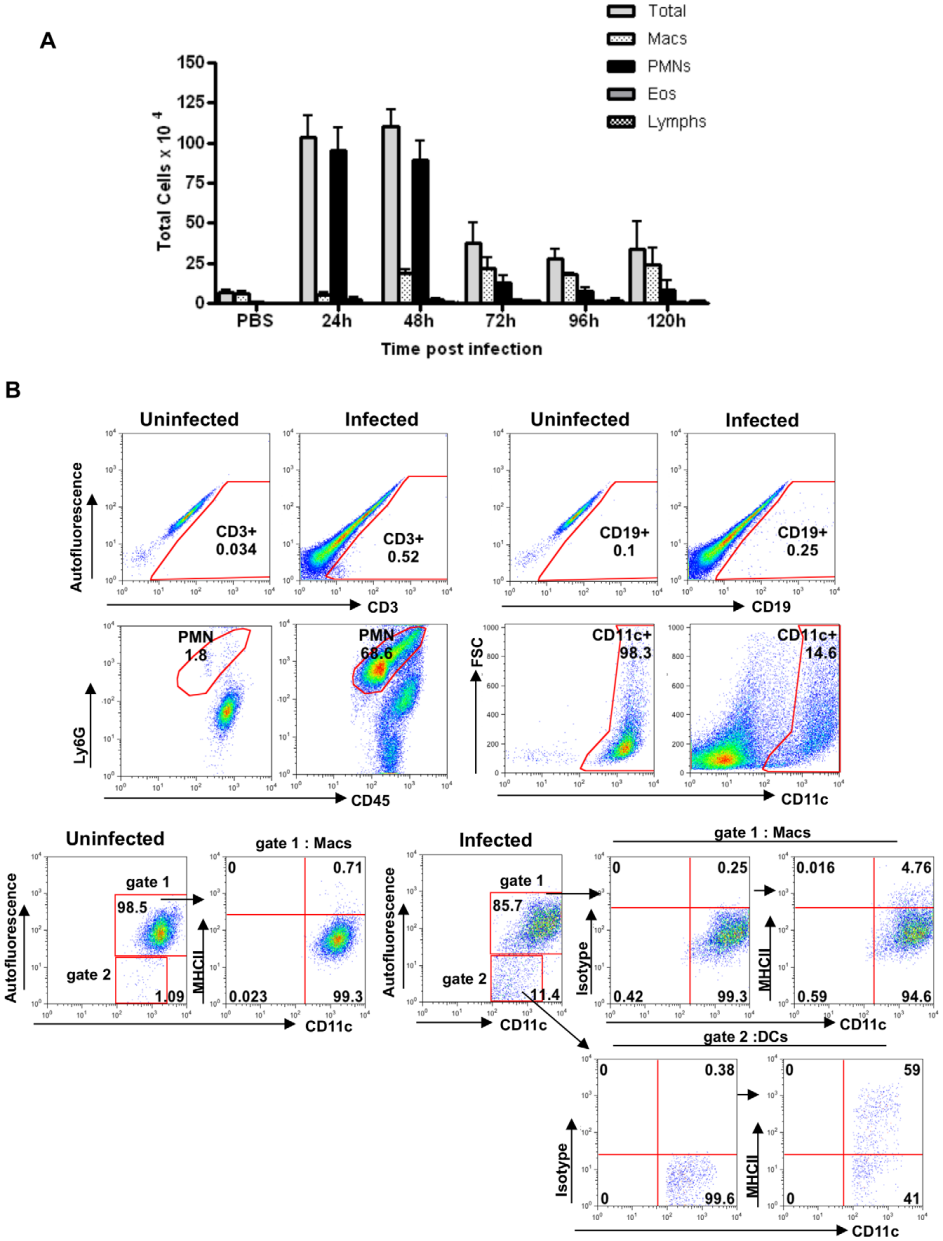
Characterization of BAL cells from after *A. fumigatus* infection. (A) Mice were infected with 50×10^6^ RC and BAL cells were collected at different time points after infection. Total and differential cell counts of BAL cells recovered from uninfected and infected mice were determined from stained cytospin slides. Macs: macrophages; PMNs: neutrophils; Eos: eosinophils and Lymphs: lymphocytes. Values shown are mean±SEM. (B) Representative flow cytometry plots showing different cell types in the BAL fluid by staining for various cell surface markers. Cells are gated on live CD45^+^ cells (leukocytes) and analyzed for CD3, CD19, Ly6G or CD11c expression. Numbers represent the percentages of specific cell types in the CD45^+^ population. Characterization of CD11c^+^ cells in the BAL fluid from uninfected or infected mice at 48 h p.i. based on autofluorescence and MHC II expression. Flow cytometry plots show that CD11c^+^ cells can be divided into highly autofluorescent macrophages (gate 1) and low autofluorescent DCs (gate 2). MHC II expression in each population was examined. Numbers represent the percentages of cells in gated populations (n = 4 mice per group).

Our next goal was to characterize the major cell types present in the alveolar space after *A. fumigatus* infection one or more of which would potentially express the molecules expressed by alternatively activated macrophages. Mice were infected with 50×10^6^ RC and BAL cells were isolated from infected and uninfected controls at various times after infection. Total and differential cell counts showed an increase in the number of polymorphonuclear neutrophils (PMNs) and macrophages, cells of the innate immune response that are important for clearing invading pathogens (Figure 2A). For the next series of experiments we used a lower dose of 10×10^6^ RC rather than a dose of 50×10^6^ RC or higher that is typically used in mortality studies in animals not treated with immunosuppressive agents [35]. Using the lower dose, at the whole lung level, a 3-4 fold increase in Arg1 expression over baseline was noted (Figure S1A). In order to characterize BAL cells further, cells were recovered from infected and uninfected controls by high volume BAL and stained for various surface markers after gating on live CD45^+^ cells (leukocytes) and analyzed for various cell types. The results showed that PMNs form the majority (68.6%) of the BAL-derived cells after 48 hours of infection as shown by Ly6G expression. However, the majority of alveolar macrophages (14.6%) remain CD11c^+^, as was also observed in the naïve mice (Figure 2B), even though the total number of CD11c^+^ cells increased significantly after infection. While ∼0.1×10^6^ total cells were recovered by BAL from naïve mice, 0.5×10^6^ cells were obtained from infected mice.

We also distinguished BAL cells based on high and low autofluorescence corresponding to macrophages and dendritic cells (DCs) respectively from uninfected and infected mice at 48 hours p.i. The majority (98.5%) of the cells from uninfected mice were CD11c^+^autofluorescence ^high^ and only1% of the cells were CD11c^+^ autofluorescence^low^. In infected mice, ∼85.7% of cells were identified as CD11c^+^ autofluorescence ^high^ alveolar macrophages and 11.4% were CD11c^+^ autofluorescence ^low^ (DCs). For further assessment, we examined the expression of MHC Class II in these two populations of cells from uninfected and infected mice. Alveolar macrophages were CD11c^+^ autofluorescence ^high^ MHC II^low^ and DCs were CD11c^+^ autofluorescence ^low^ MHC II^high^ (Figure 2B). Based on these results, we used autofluorescence ^high^ CD11c^+^ cells corresponding to alveolar macrophages for further experiments.

### *A. fumigatus* infection-induces CD11c^+^ autofluorescence^high^ Arg1- expressing alveolar macrophages

**Figure 3:**
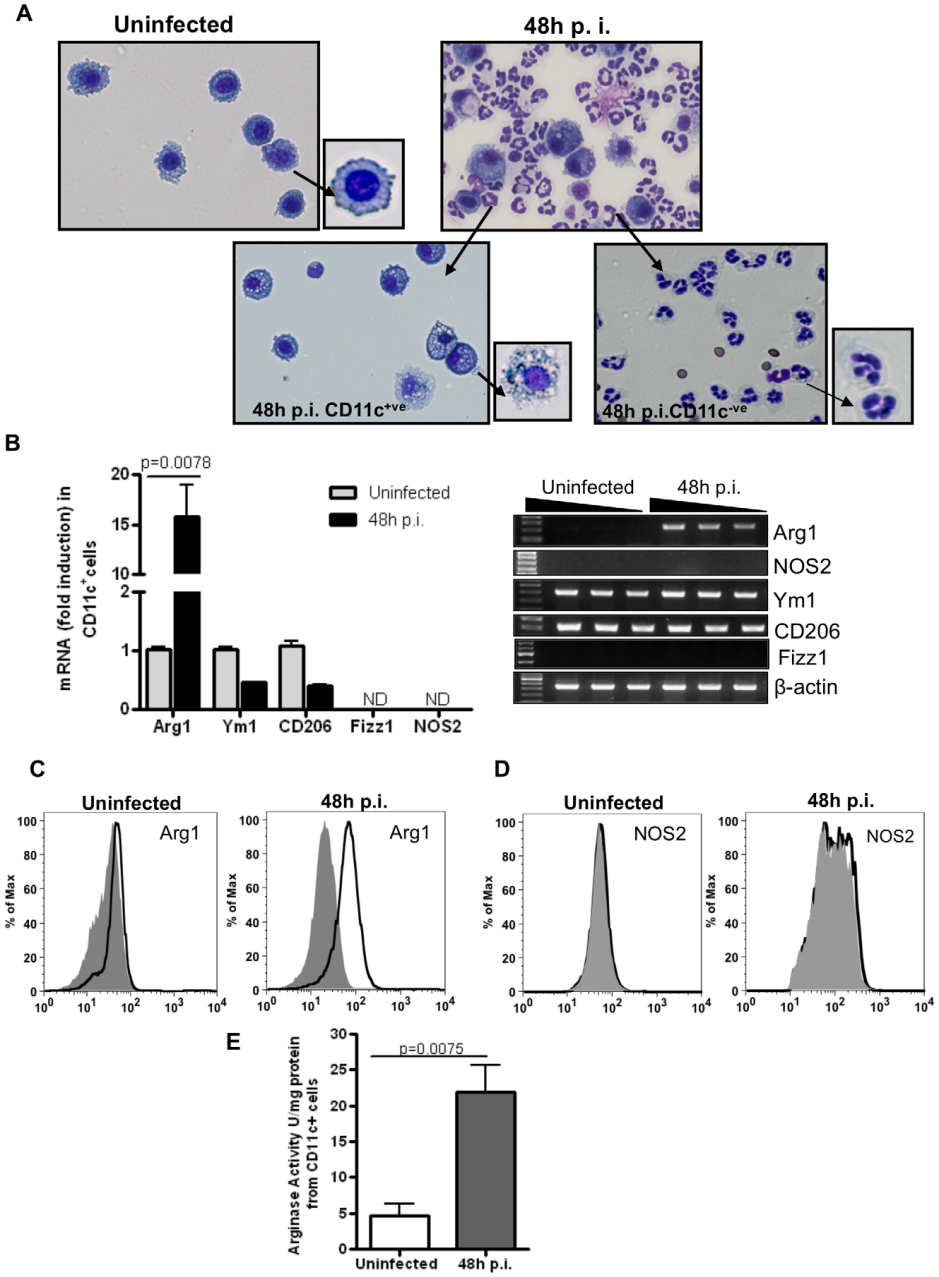
Identification of CD11c^+^Arg1-expressing alveolar macrophages after *A. fumigatus* infection. (A) Mice were infected with 10×10^6^ RC and cells in the BAL fluid were recovered from uninfected and infected mice. Stained cytospins of CD11c**^+^** and CD11c^-^ fractions 48 hours p.i. CD11c^+^ macrophages before and after infection exhibited different morphology when compared to those isolated from naïve controls. (B) mRNA expression of AAM markers measured by quantitative (left panel) and semi-quantitative RT-PCR (right panel) in BAL CD11c**^+^** cells isolated 48 hours p.i. The fold increase shown is relative to genes expressed in CD11c**^+^**cells from the uninfected group after normalization to Gus-β. Values shown are mean±SEM. For semi-quantitative RT-PCR, β-actin expression was used as an internal control. Data shown were generated using RNA from 1 mouse (n = 4) with the PCR products in the different lanes generated with increasing dilutions of cDNA. The experiment was repeated three times with similar results. (C) Arg1 and (D) NOS2 expression in the BAL CD11c**^+^** cells was examined at 48 hours p.i. by flow cytometry using intracellular staining techniques. Gray (filled) and black (open) histograms denote staining with isotype control and specific anti-Arg1 or anti-NOS2 antibody respectively (n = 4–6 mice in each group). The frequency of Arg1 expression in uninfected and infected cells was 2.2% and 52% respectively while that of NOS2 was 0.68% and 2.54% in the same cells. (E) Arginase activity expressed as U/mg protein was measured in protein extracts made from CD11c**^+^** cells isolated by BAL from PBS-treated uninfected controls or fungus-infected mice at 48 hours p.i.

Next, we sought to determine whether alveolar macrophages expressed Arg1 but not NOS2 upon infection by *A*. *fumigatus*. Mice were infected with 10×10^6^ RC and CD11c^+^ cells were recovered by BAL 48 hours p.i. In addition to the increase in the number of CD11c^+^ cells in the alveolar space after fungal infection, the alveolar macrophages in the infected mice also acquired a distinct morphology and were found to be more vacuolated as compared to those in naïve mice (Figure 3A). Similar results were obtained in C57BL/6 mice (data not shown). The CD11c^–^ fraction comprised mainly PMNs (Figure 3A). To determine the nature of these macrophages, Arg1 expression was examined in purified CD11c^+^ cells. Based on their high autofluorescence and morphology, the cells were uniformly identified as macrophages. As shown in Figure 3B, a robust increase in Arg1 expression was noted in these CD11c^+^ cells suggesting development of AAM-like cells in the infected lungs.

We assessed expression of other AAM-associated markers in the CD11c^+^ cells. Arg1 was the only gene whose expression was upregulated in the CD11c^+^ BAL cells from infected mice as compared to expression in cells isolated from the controls (Figure 3B). However, Ym1 and CD206, genes also associated with AAMs [7], [8], [10], were found to be constitutively expressed in alveolar macrophages isolated from uninfected mice (Figure 3B). Fizz1/RELM-α was not detected in these cells whether the cells were isolated from infected or uninfected mice. It has been previously shown that the basal levels of expression of Ym1 and Fizz1 differ in macrophages isolated from different tissues presumably due to differential stimulation by the microenvironments they reside in [36]. The lack of Fizz1 expression in the CD11c^+^ cells suggested that the increased expression of this molecule observed in the lungs of infected mice was contributed by tissue resident cells such as epithelial cells and eosinophils [37]. At this early time point after infection, eosinophil infiltration is quite low making it unlikely that these cells contributed much to Fizz1 expression in the infected lungs which makes epithelial cells the likely source of this molecule. The signature marker for CAMs, NOS2, was barely detectable in the CD11c^+^ population (Figure 3B). In the lungs too, NOS2 was not detected at any time point after infection (Figure 1, panels B and C and Figure S1, panels A and B). Thus, macrophages expressing Arg1, Ym1 and CD206 were the dominant alveolar CD11c^+^ cells early after *A*. *fumigatus* infection.

We also examined the expression of Arg1 and NOS2 by intracellular staining of CD11c^+^ cells purified from infected mice after 48 hours and compared with expression in cells recovered from uninfected mice. It was clear in these experiments that Aspergillus infection induces robust Arg1 expression but not NOS2. Compared to >50% of the cells expressing Arg1 at 48 after infection (Figure 3C), only 2.5% of the cells were found to be NOS2^+^ by intracellular staining techniques (Figure 3D). Further, we also assayed arginase activity in CD11c^+^ cells isolated by BAL from infected and uninfected mice. As shown in Figure 3E, significantly higher arginase activity was evident when cells were isolated from infected mice, further providing the evidence that alveolar macrophages after Aspergillus infection have a predominance of alternatively activated phenotype.

### CD11c^+^Arg1-expressing macrophages isolated after *A. fumigatus* infection carry fungal load

**Figure 4:**
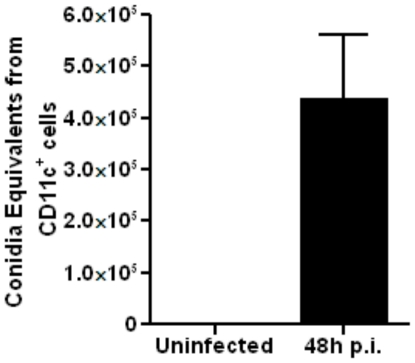
CD11c^+^Arg1 expressing AAMs isolated after *A. fumigatus* infection carry fungal load. Mice were infected with 10×10^6^ RC and CD11c**^+^** cells were isolated by BAL from PBS-treated uninfected controls or fungus-infected mice at 48 hours p.i. Fungal uptake in the BAL CD11c**^+^** cells was assessed by quantitative PCR of DNA corresponding to fungal 18S rRNA and expressed as Conidia Equivalents/lung (n = 4 mice in each group). Values shown are mean±SEM.

It was previously shown that the lack of NOS2 expression has no effect on the killing of fungal conidia by alveolar macrophages [38]. Since the majority of the alveolar macrophages expressed Arg1 after fungal infection (Figure 3, panels B and C), we were curious whether the CD11c^+^ cells isolated and purified from BAL fluid had the ability to phagocytose fungal conidia. Mice were infected with 10×10^6^ RC and CD11c^+^ cells were recovered by BAL 48 hours p.i. Fungal load was measured by quantitative PCR of fungal DNA corresponding to fungal 18S rRNA and expressed as conidia equivalents in CD11c^+^ cells (Figure 4). The data suggested that alveolar macrophages, a large fraction of which expresses Arg1 after fungal infection, can efficiently phagocytose conidia.

### *A. fumigatus*-induced Arg 1 expression is partially dependent on IL-4Rα/STAT-6 signaling

**Figure 5:**
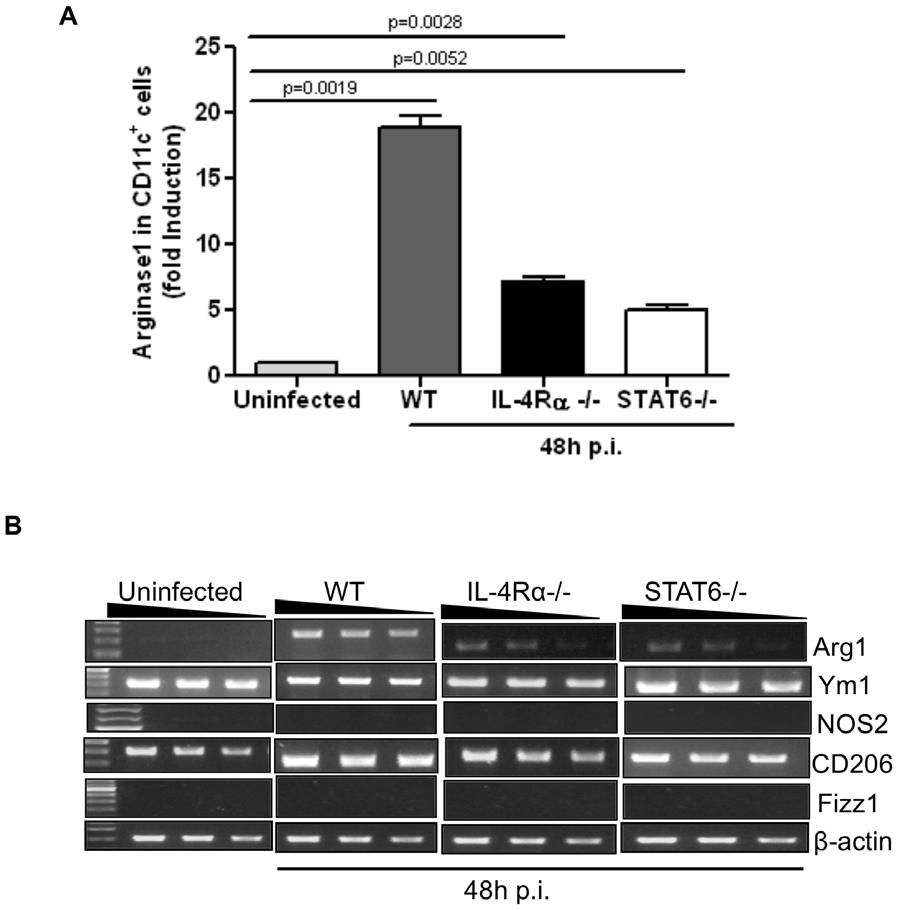
IL-4Rα/STAT6 partly controls Arg1 expression in alveolar macrophages isolated from *A. fumigatus*-infected mice. WT, IL-4Rα^-/-^ and Stat6^-/-^ mice were infected with 10×10^6^ RC and CD11c**^+^** cells were isolated by BAL at 48 hours p.i. (A) Quantitative RT-PCR was performed to measure Arg1 mRNA expression in CD11c**^+^** cells from infected WT, IL-4Rα^-/-^ and Stat6^-/-^ at 48 hours p.i. and the fold increase shown are relative to that in CD11c**^+^** cells from uninfected group. Values shown are mean±SEM (B) Semi-quantitative RT-PCR analysis of expression of indicated genes CD11c^+^ cells isolated from uninfected and infected mice. The data were obtained using RNA isolated from the cells of one mouse with the bands in the 3 lanes in each group depicting PCR products obtained with increasing dilution of cDNA. Similar results were obtained in two independent experiments (n = 4–6 mice in each group).

AAMs or M2 macrophages can be elicited *in vitro* in the presence of Th2 cytokines such as IL-4 and IL-13 [4], [39]. The development and maintenance of AAMs *in vivo* involve IL-4Rα/STAT6 signaling, the common signaling pathway for IL-4 and IL-13 [20], [24] and the induction of AAM-specific genes was also shown to be dependent on this signaling axis [40], [41], [42]. We therefore explored the validity of this pathway in the expression of Arg1, the prototypical marker of AAMs, in our study. Mice deficient in IL-4Rα or STAT6 were infected with 10×10^6^ RC along with WT BALB/c controls. CD11c^+^ BAL alveolar macrophages were purified from WT, IL-4Rα^-/-^ and STAT6^-/-^ mice and Arg1 expression was measured. Arg1 was found to be reduced by ∼50% in the BAL macrophages isolated from IL-4Rα^-/-^ and STAT6 ^-/-^ mice as compared to that in cells from WT mice (Figure 5A). The fact that Arg1 expression was not completely ablated in cells deficient in signaling downstream of IL-4Rα suggested that factors other than STAT6, triggered by fungal surface molecules contribute to the maximal level of Arg1 expression observed in BAL CD11c^+^ cells isolated from WT mice. The expression of other AAM-associated genes such as Ym1 and CD206 remained unaffected in cells from IL-4Rα^-/-^ and STAT6^-/-^ mice (Figure 5B). These data also suggested that the basal level of Ym1 or CD206 expression in alveolar macrophages is not driven by IL-4Rα/STAT6 signaling. However, similar to the ability of IL-4 and IL-13 to induce the alternatively activated phenotype in macrophages in the context of helminth infections [7], fungus-induced Arg1 was also found to be at least partially dependent on IL-4Rα/STAT6.

### Dectin-1 and MyD88 involvement in Arg1 expression and fungal clearance

**Figure 6:**
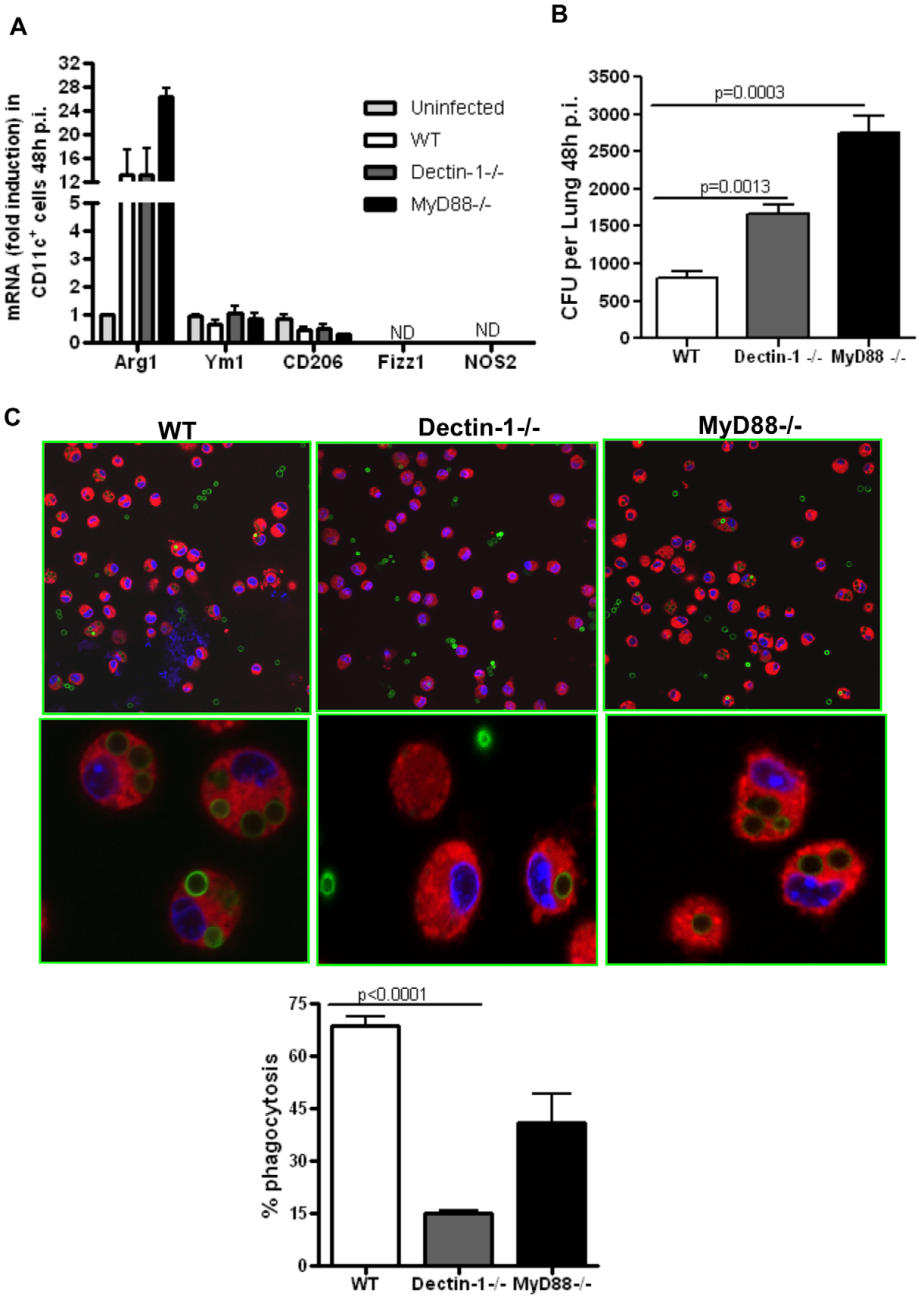
Dectin-1- or MyD88-deficiency in alveolar macrophages does not affect Arginase1 expression but impairs fungal clearance. (A) WT, Dectin-1^-/-^ and MyD88^-/-^ mice were infected with 10×10^6^ RC and CD11c^+^ cells were isolated by BAL at 48 hours p.i. Quantitative RT-PCR was performed to measure Arg1 mRNA expression in CD11c^+^ cells from infected WT, Dectin-1^-/-^ and MyD88^-/-^ and the fold increase shown are relative to that in CD11c^+^ cells from uninfected group. Values shown are mean±SEM (B) Fungal burden expressed as CFU per lung was measured in lungs harvested from WT, Dectin-1^-/-^ and MyD88^-/-^ mice at 48 hours p.i. Values shown are mean±SEM. Data shown are representative of two independent experiments (n = 6-8 mice in each group). (C) Phagocytosis of FITC-labeled *A. fumigatus* conidia by CD11c^+^ alveolar macrophages as examined by confocal microscopy. Images show overlay of FITC (green for conidia) and Hoechst stain (blue for nuclei) and Cell tracker (red for cell cytoplasm). The upper panel shows images captured at 60X in single optical plane and the lower panel shows 3X digital zoom images. Quantification of conidia present intracellularly was done using Metamorph and results are expressed as percentage of cells with FITC-conidia. Labeled conidia were easily identified in the macrophages isolated from both WT and MyD88-deficient mice but were rare in cells isolated from Dectin-1^-/-^ mice.

Given that Arg1 expression was not completely dependent on the IL-4Rα/STAT6 signaling pathway, we were curious whether pattern recognition receptors on the macrophages contributed to the expression of these molecules in infected mice. In this regard, Dectin-1, the pattern recognition receptor that binds β-glucan expressed on fungal cell walls [29], [31], [43] and MyD88, the essential adaptor molecule for signaling downstream of most TLRs [44], were the key candidates. Dectin-1 has been shown to be important for antifungal defense [32], [33], [35], [45] and MyD88 is also utilized once conidia germinate to hyphae [25], [29], [46]. An important role for Dectin-1 in uptake of β-glucan-expressing zymosans in phagosomes of macrophages was demonstrated and Dectin-1 was shown to augment TLR/MyD88-induced pro-inflammatory cytokines in the zymosan-exposed macrophages [47]. We first examined Arg1 expression in BAL CD11c^+^ cells isolated from WT, Dectin-1^-/-^ and MyD88^-/-^ mice. As shown in Figure 6A, Aspergillus- induced Arg1 expression in alveolar macrophages was independent of these signaling pathways. The expression of other AAM-associated genes such as Ym1 and CD206 remained unaffected in cells from Dectin-1^-/-^ and MyD88^-/-^ mice (Figure 6A). We next investigated fungal burden in WT, Dectin-1^-/-^ and MyD88^-/-^ mice at 48 hours p.i. As shown in Figure 6B, fungal burden was 2-3-fold more in the lungs of both Dectin-1^-/-^ and MyD88^-/-^ mice.

With the observation that Dectin-1 and MyD88 deficiency results in higher fungal burden in the lung when compared to wild type mice, we further investigated phagocytosis of fungal conidia by alveolar macrophages isolated from WT, Dectin-1^-/-^ and MyD88^-/-^ mice using FITC-labeled live conidia. We examined the presence of phagocytosed conidia in live cells from WT, Dectin-1^-/-^ and MyD88^-/-^ mice by confocal microscopy. While alveolar macrophages isolated from either WT or MyD88^-/-^ showed presence of FITC-conidia inside the cells (Figure 6C), fewer macrophages from Dectin-1-deficient mice showed labeled conidia inside the cells in line with previous observations establishing a role for Dectin-1 in phagocytosis by sensing β-glucan [47]. Although Dectin-1 has been directly associated with phagocytosis [47], the slightly lower efficiency of the MyD88-deficient cells in phagocytosis as compared to WT cells may have been due to the inability of Dectin-1 to collaborate with the TLR pathway in the MyD88-deficient cells. These data showed that unlike IL-4Rα/STAT6, Dectin-1 and TLR/MyD88 do not regulate Arg1 expression but nonetheless play an important role in fungal clearance given their function in sensing fungus-expressed molecules and phagocytosis and induction of inflammatory responses [47].

### Depletion of macrophages decrease pulmonary clearance of *A. fumigatus*

**Figure 7:**
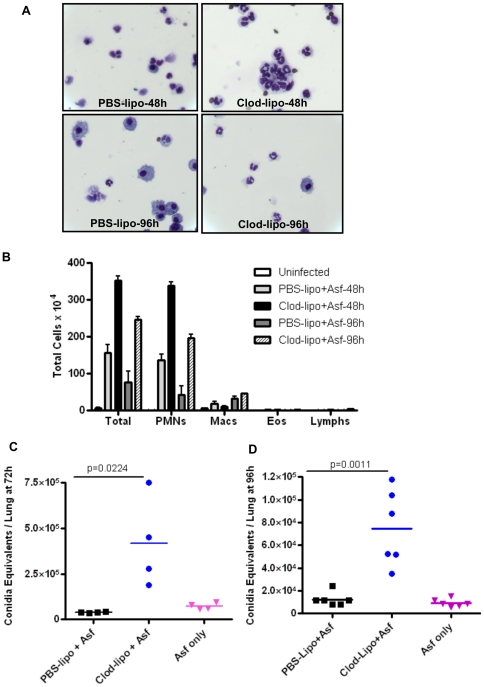
Clodronate-mediated depletion of alveolar macrophages increases *A. fumigatus* burden in the lung. Mice were treated with clodronate-filled liposomes or PBS-liposomes intratracheally at 48 hours prior to *A. fumigatus* infection and then infected with 50×10^6^ RC. (A) Cytospins of cells present in BAL fluid recovered from PBS-liposome or clodronate-liposome treated mice at 48 and 96 hours p.i. (B) Total and differential cell counts of BAL-derived cells from PBS-liposome and clodronate-liposome treated mice at 48 and 96 hours p.i. Results shown are mean±SEM. (C and D) Fungal burden was assessed by quantitative PCR of DNA corresponding to fungal 18S rRNA and expressed as Conidia Equivalents/lung. The experiment was repeated twice with similar results (n = 4–8 mice in each group). Values shown are mean±SEM.

Since our investigations showed that Aspergillus infection promotes the development of Arg1-expressing alveolar macrophages and at none of the time points we could detect NOS2, we asked whether these cells were important in fungal clearance in the infected host. One of the strategies used to deplete macrophages is making use of clodronate-loaded liposomes that selectively deplete monocytes, macrophages but not lymphocytes or neutrophils [48], [49]. Although alveolar dendritic cells (DCs) (but not interstitial DCs) are also at least partially depleted by clodronate-liposomes [50], this was not of concern to us for two reasons. First, macrophages are significantly more numerous and the key phagocytic cells in the alveolar space in naïve mice and second, our objective was to determine effects on fungal burden and not adaptive immune responses. Clodronate-loaded liposomes or control PBS-filled liposomes were administered intratracheally 48 hours prior to fungus infection and mice were subsequently infected with 5 fold more RC (50 million), a high fungal dose that is used in mortality studies [35]. When compared with PBS-liposome group, mice that received clodronate-liposomes showed reduced numbers of macrophages (Figure 7A) both at 48 and 96 hours after fungus infection. Further, we measured the total and differential counts in the BAL cells in these two groups. We observed an impressive increase in total cell counts in the clodronate group at both time points due to compensatory increase in PMNs which caused a higher PMN/macrophage ratio due to depletion of macrophages but increase in PMNs (Figure 7B). Fungal burden was compared between PBS-liposome and clodronate-liposome groups as well as in infected mice without liposome administration (additional control) at 72 and 96 hours p.i. As shown in Figure 7, panels C and D, when clodronate–liposomes were administered prior to intratracheal administration of 50×10^6^ RC, fungal burden was significantly higher at 72 and 96 hours p.i. showing that alveolar macrophages in fungus-infected mice are important for reducing fungal burden in the lungs. Remarkably, the increased numbers of PMNs were unable to control fungal burden.

Taken together, these results showed that alveolar macrophages, the majority of which assume an alternative phenotype with the induction of Arg1 in response to Aspergillus infection with no detectable expression of NOS2, play an important role in pathogen clearance immediately after fungal infection.

## Discussion

**Figure 8:**
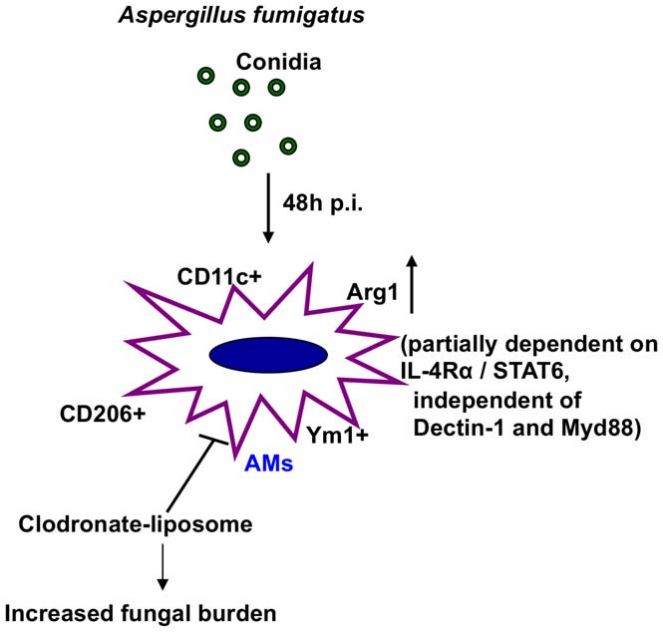
Alveolar macropjages expressing Arginase 1 dominate after *A. fumigatus* infection and role in fungal clearance. Infection by *A. fumigatus* rapidly induces Arg1 expression in alveolar macrophages. Arg1 expression is partly dependent on the IL-4Rα/STAT6 signaling axis. Furthermore, Arg1 expression is independent of Dectin-1 and MyD88 signaling pathways. Clodronate-mediated depletion of alveolar macrophages prior to fungal infection results in increased fungal burden in the lungs.

In immunocompetent healthy individuals, inhaled spores of *A. fumigatus* are rapidly cleared off in which innate immunity is believed to be sufficient for clearing the fungus [3]. However, in immunocompromised patients, inhaled conidia germinate and invade the parenchyma. In patients with cystic fibrosis or severe asthma, impaired fungal clearance induces allergic disease termed allergic bronchopulmonary aspergillosis (ABPA). Efficient and prompt fungal clearance is therefore of utmost importance to prevent fungus-induced disease. However, the mechanisms underlying fungal clearance are not well understood. Our study for the first time shows an important role of alveolar macrophages in fungal recognition and clearance immediately after fungal infection. Fungal infection rapidly induced Arg1 expression in alveolar macrophages, which was also true for tissue macrophages (not shown). Besides Arg1, the alveolar macrophages in fungus-infected mice were also found to express other AAM-associated molecules such as Ym1 and CD206. Arg1 induction upon fungal infection was partially dependent on the IL-4Rα/STAT6 signaling axis. The β-glucan receptor, Dectin-1, was found to play an important role in the phagocytosis of Aspergillus by alveolar macrophages and its absence increased fungal burden in the lungs of the infected mice. Lack of MyD88, the adaptor downstream of most TLRs, known to recognize Aspergillus and cooperate with Dectin-1 for the induction of inflammatory responses [47], also impaired fungal clearance although neither Dectin-1 nor MyD88 contributed to Arg1 expression in the infected macrophages. Depletion of alveolar macrophages increased fungal burden in the lungs of mice despite of increased influx of neutrophils in the alveolar space. Figure 8 illustrates the key findings in our study.

Macrophages constitute one of the most important cells of innate immunity with versatile functions. Recently, heterogeneity in macrophage phenotype and function has been well recognized similar to that noted for T cells [7]. Macrophages have been subdivided into two broad categories, M1 and M2. M1 macrophages express NOS2 and reactive oxygen and nitrogen intermediates and are IL-12^high^ and IL-10^low^. M2 macrophages express Arg1 along with a host of other molecules such as Ym1 and Fizz1 and are IL-12^low^ and IL-10^high^. Depending on context, variability in expression of these molecules is increasingly being noted and the M2 category has been broadened to include all additional subtypes [8], [51]. Classical or M1 macrophages are crucial for killing pathogens and tumor cells [7], [52]. M2 macrophages/AAMs have been associated with both adverse and beneficial effects in interactions of the host with various pathogens. For example, a recent study showed that Arg1, the key enzyme expressed by AAMs, can be detrimental during infections by intracellular pathogens such as *T. gondii* and *M. bovis* [11]. In this study, Arg1, which uses the same substrate, L-Arg, as NOS2, was found to help survival of the intracellular pathogens due to a decrease in NO production [11]. However, in the context of worm infections, where AAMs have been studied the most, these cells have been largely associated with beneficial effects in the infected host. For example, recent studies have highlighted an important role for Fizz1 expressed by AAMs in suppressing Th2 responses and downregulating inflammation and fibrosis in mice infected with *S. mansoni* [22], [23] and Arg1 was also associated with similar suppressive functions [21]. The protective role of AAMs in schistosomiasis was also shown to involve downregulation of harmful Th1 inflammatory responses and AAM induction was essential for survival [53]. Clodronate-mediated removal of macrophages with AAM phenotype in the intestines of mice infected with *N. brasieliensis* impaired smooth muscle contractility and increase in thickness and worm expulsion [20], [54]. In the case of infection by the worm *Brugia malayi*, absence of AAMs resulted in increased neutrophilia and reduced eosinophilia [55]. In this regard, the AAMs were shown to phagocytose apoptotic neutrophils. Thus, at the present time there exists a significant body of literature on AAM characterization and function in the context of chronic infections, particularly in the context of helminth infections, and for the most part this type of host response has been found to be beneficial. However, fewer studies have studied these cells early after infection. In the RSV infection study, AAMs expressing Th2 cytokines were detected around 4 days p.i. [13]. Compared to that observed in WT mice, infection of IL-4Rα^-/-^ mice by RSV that impaired AAM development caused worse lung pathology and thus a protective role of these cells was suggested [13].

In our study, the experiments performed to address the relevance of alveolar macrophages in Aspergillus infection suggest a protective role of these cells in the context of fungal infection although we could not address the specific role of the Arg1-expressing AAM-type population due to our inability to selectively deplete them. Fungus-induced Arg1 expression in alveolar macrophages was also rapidly induced as early as 6 hours after infection and at none of the time points tested did we detect appreciable NOS2 expression unlike in mice infected with *K. pneumoniae* that showed brisk NOS2 expression (Figure 1). The decline of Arg1 mRNA levels after 96 hours of infection was in toe with fungal clearance. Furthermore, in phagocytosis assays, macrophages isolated from WT mice showed significantly higher phagocytosis as compared to those from Dectin-1-deficient mice (Figure 6). In the in vivo setting, we also detected fungal DNA corresponding to 18S rRNA in CD11c^+^ cells isolated from infected mice (Figure 4). Finally, depletion of alveolar macrophages using clodronate-liposomes increased fungal burden in the lung at multiple time points (Figure 7). Collectively, these results suggest that a population of alveolar macrophages with a predominance of alternatively activated phenotype is beneficial in rapid clearance of fungi from infected lungs.

The induction of various AAM-associated genes such as Arg1, Ym1 and Fizz1 in the majority of studies has been found to require the IL-4Rα/STAT6 signaling axis [12], [54], [56], [57], [58]. However, exceptions have been noted as during infection by *T. gondii* or *M. bovis* where TLR-mediated signaling is required [11] or during development of trypanosomiasis where IL-10-mediated mechanisms were invoked [59]. We show that Arg1 expression is reduced but not eliminated in IL-4Rα^-/-^ or STAT6^-/-^ mice (Figures 5). It is possible that functional cooperation between IL-4Rα/STAT6 and additional pathways promotes maximal Arg1 expression in alveolar macrophages after infection with *A. fumigatus*. In studies of infection by *Fasciola hepatica* and *S. mansoni*, the secreted antioxidant, peroxiredoxin (Prx), was shown to induce Ym1-expressing AAMs, which enhanced the secretion of IL-4, IL-5 and IL-13 from naïve CD4^+^T cells [60]. However, any such possibility in our study remains to be determined. Cell surface molecules such as Dectin-1 and TLRs are integral to fungal recognition; however the role of these molecules in the induction of markers commonly associated with the AAM phenotype such as Arg1 has not been previously studied. Our data show that unlike IL-4Rα/STAT6, Dectin-1 or MyD88 do not contribute to Arg1 expression suggesting a division of labor between different cell surface molecules with respect to pathogen recognition and uptake and induction of intracellular molecules such as Arg1.

The localization of Dectin-1 to phagosomes and its important role in phagocytosis of zymosan particles expressing β-glucan, the ligand of Dectin-1, was previously shown [47]. This study also showed the collaborative efforts of Dectin-1, exerted via its ITAM (immunoreceptor tyrosine-based activation) motif and TLR2 via MyD88 signaling in macrophages in both phagocytosis and expression of pro-inflammatory cytokines and reactive oxygen species [47]. As many subsequent studies have shown, fungal pathogens like *Aspergillus, Pneumocystis* and *Candida* utilize TLRs and Dectin-1 to infect macrophages and neutrophils [25], [29], [31], [32], [33], [35], [43], [45], [46], [51]. Therefore, we believe that the increased burden noticed in the Dectin-1^-/-^ and MyD88^-/-^ mice after 48 hours of *A. fumigatus* infection is due to the lack of the collaborative effort between the two signaling pathways in the infected macrophages. Given that lack of MyD88 did not significantly impair fungal uptake by the alveolar macrophages but still increased fungal burden, it is likely that in the absence of MyD88, inadequate production of proinflammatory cytokines that are required to kill phagocytosed microbes accounts for the delay in fungal clearance in these mice.

So, how might Arg1 induced by Aspergillus cause increased fungal clearance? In the *H. polygyrus* infection study, AAMs were associated with impairment of larval parasite health and mobility and worm expulsion that was dependent on Arginase expression [24]. Similarly, Arg1 was found to be crucial for suppression of Th2 responses in mice infected with *S. mansoni* [21]. How Arg1 might contribute to the suppressive functions of AAMs at early or late time points after infection is currently not understood. Metabolism of L-Arg by Arg1, the major arginase activity in the body [61], generates L-ornithine and urea. L-ornithine is metabolized by ornithine decarboxylase to the polyamine putrescine, which is further converted to other polyamines. L-ornithine is also metabolized in the mitochondria via successive steps to L-proline, which is essential for the synthesis of many structural proteins, including collagen [62]. Competition of Arg1-expressing macrophages with myofibroblasts for the substrate L-Arg causing less collagen production by the myofibroblasts has been suggested as one possible mechanism for suppression of fibrosis by AAMs induced after infection by *S. mansoni* [21]. In our study, none of these mechanisms is relevant since we have studied fungal clearance at an early time point before the induction of adaptive immunity. However, one important consideration is competition for L-Arg between the germinating fungal spores and the AAMs. The Aspergillus species, *A. nidulans*, was shown to utilize L-Arg as a source for nitrogen and carbon employing arginase enzymes [63], [64]. It is likely that Arg1-expressing macrophages competitively deprive the fungus of L-Arg and compromise spore viability. Interestingly, in human neutrophils, L-Arg depletion by Arg1 localized to phagolysosomes was recognized as a novel mechanism of anti-fungal activity against *Candida albicans* [34]. It is a well recognized phenomenon that myeloid cells deplete phagosomes of critical nutrients required for survival of phagocytosed microbes [65]. The microbes, in turn, try to compensate by upregulating expression of genes to adapt to the host microenvironment. The fungi *C. albicans* and *Saccharomyces cerevisiae* were shown to upregulate expression of genes associated with Arginine biosynthesis in human neutrophils [66]. Taken together, the finding of a role for Arg1 constitutively expressed in human neutrophils in defense against *C. albicans* [34], the dependence on L-Arg by fungi as an essential nutrient source [63], [64], and our collective data of the role of alveolar macrophages with AAM phenotype in Aspergillus uptake and clearance provide logical explanations for why the host would attempt to rapidly induce Arg1 in the infected lung macrophages. Since Aspergillus is a ubiquitous pathogen and the host has to fight this battle with the fungus continuously, it makes more sense to express Arg1 rather than NOS2 to deplete L-Arg since constant generation of NO via NOS2 activity would be deleterious to lung health. Thus, alveolar macrophages with prevalence of AAMs following *A. fumigatus* infection play an important role in innate immune response.

## Materials and Methods

### Ethics statement

All animal work was conducted in accordance with guidelines issued by the Institutional Animal Care and Use Committee of the University of Pittsburgh and our approved protocol ID is 1005244. The Institutional Animal Care and Use Committee of the University of Pittsburgh is in compliance with Public Health Service (PHS) Policy on Humane Care and Use of Laboratory Animals when using live, vertebrate animals. PHS Policy incorporates U.S. Government Principles, the Guide for the Care and Use of Laboratory Animals, and the Report of the American Veterinary Medical Association (AVMA) Panel on Euthanasia. Mice were bred and maintained in the Department of Laboratory Animal Resources (DLAR) at the University of Pittsburgh. Mice were maintained in pathogen free environment and kept in sterile filtered top cages, maintained on 12 h dark/light cycle.

### Mice

Male 6–8 weeks old BALB/c IL-4Rα^-/-^ and STAT6^-/-^ mice were purchased from the Jackson Laboratories. MyD88^-/-^ [44] and Dectin-1^-/-^ mice [32] on the BALB/c background were bred at the animal facility at the University of Pittsburgh.

### Infection by *A. fumigatus*

*A. fumigatus* isolate 13073 (American Type Culture Collection) was grown on Potato Dextrose Agar (PDA) media for 5–7 days at 37°C in a culture flask. Conidia were harvested with 50 ml of sterile PBS containing 0.1% Tween-20. The harvested conidia were then passed through sterile 40 µm strainer and counted on a hemacytometer. Mice were infected with 10–50×10^6^ resting conidia (RC) suspended in 50 µl of sterile PBS and administered intratracheally after anaesthetizing mice with isofluorane.

### BAL

Cells were collected by bronchioalveolar Lavage (BAL) from naïve and infected mice after high volume lavage with 1ml 1x PBS successively 10 times. BAL cells were subjected to CD11c purification using magnetic beads (Miltenyi Biotech) against mouse-specific CD11c described previously [67], [68], [69]. Cytospins of cells were stained with Hema-3 reagents (Fisher Scientific) according to the manufacturer's recommendations.

### Clodronate-Liposome mediated depletion of alveolar macrophages

Macrophages were depleted using liposomes containing clodronate. Clodronate was incorporated into liposomes as described previously [49]. Mice were given 100 µl (25 mg/mouse) of PBS-liposome or clodronate-liposome intratracheally 48 hour prior to administration of *A. fumigatus* infection after anesthetizing the mice with the isofluorane.

### RT-PCR and Real time quantitative PCR

Total RNA was isolated from whole lung samples or from purified cell populations at various times following *A. fumigatus* infection. RNA was isolated from TRIzol (Invitrogen Life Technologies) suspended samples using RNeasy Mini kit from Qiagen. The purified RNA was subsequently used for cDNA preparation using a Reverse Transcriptase-PCR kit (Applied Biosystems). The following mouse-specific oligodeoxynucleotides were used for RT-PCR analysis: Arginase1- FP 5′ATG GAA GAG ACC TTC AGC TAC 3′, RP 5′GCT GTC TTC CCA AGA GTT GGG 3′;Chi3l3- FP 5′ GGG CAT ACC TTT ATC CTG AG 3′,RP 5′ CCA CTG AAG TCA TCC ATG TC 3′; NOS2 – FP 5′CCCTTCCGAAGTTTCTGGCAGC 3′, RP5′GCGTGTCAGAGCCTCGTGGCTTTGG 3′; Fizz-1 FP 5′ TCC CAG TGA ATA CTG ATG AGA 3′, RP 5′ CCA CTC TGG ATC TCC CAA GA 3′, CD206- FP 5′ GCA AAT GGA GCC GTC TGT GC 3′, RP 5′ CTC GTG GAT CTC CGT GAC AC 3′, β-actin FP 5′ TGGAATCCTGTGGCATCCATGAAAC 3′, RP 5′TAAAACGCAGCTCAGTAACAGTCCG 3′. For semi-quantitative analyses, all reactions involved 30 PCR cycles. After amplification, the samples were separated on 2% molecular biology grade agarose gels containing ethidium bromide and bands were visualized and photographed using UV transillumination. For quantitative (real time) RT-PCR, specific TaqMAN gene expression assays were obtained from Applied Biosystems which included those for Arginase1 (Mm01190441_g1), Chi3l3 (Mm00657889_mH), Fizz1 (Mm00443109_m1), NOS2 **(**Mm00440488_m1), Gus-β (Mm00446953_m1), CD206 (Mm01329362_m1) and Real time RT-PCR was performed on cDNA using TaqMAN assay. Reactions were run in a real time PCR system (ABI 7900 HT; Applied Biosystems). The results were analyzed using SDS 2.2.2 software and samples were normalized to Gus-β. Fold induction was calculated over PBS treated or untreated controls unless otherwise indicated.

### Western blotting

Non-denaturing cell lysis buffer containing 1% Triton (Cell Signaling) was used to prepare total lung extracts. Western blotting techniques were used to analyze equal amounts of protein as described previously [67]. Membranes were probed with monoclonal antibodies against YM1 (Stem Cell Technology) at a 1/1000 dilution. After stripping, the blots were probed with anti-β-actin (Jackson laboratory) to confirm equal protein loading. The intensity of the YM1 signal was quantified relative to that of β-actin using image J software.

### Fungal Burden

Fungal burden was calculated by isolating DNA from infected lung tissue using Epicentre Yeast DNA isolation kit. Real time PCR was done with DNA as the template using Aspergillus-specific oligonucleotides and the results were analyzed according to a previously described method [70]. Fungal burden was expressed as Conidia Equivalents/lung (CE/lung). Fungal burden was also measured by plating lung homogenates on PDA plates and colonies were counted. Fungal burden was expressed as colony forming units/lung (CFU/lung).

### Arginase Activity

For assay of arginase activity, total lung or cell extracts were made using 1x cell lysis buffer (Cell Signaling). Arginase activity was measured using the DARG-200 kit (Bioassay Systems). Protein concentration was measured using the BCA kit (BioRad Laboratories) and arginase activity was expressed as U/mg Protein.

### Intracellular staining and flow cytometric analysis

Staining for cell surface expression of CD45, CD3, CD19, CD11c, Ly6G, and MHC II was carried out using specific antibodies as described previously [67]. Intracellular staining was done according to the manufacturer's suggestions (Cytofix/Cytoperm, BD Pharmingen). For Arg1 staining, purified mouse anti-Arg-1 antibody (BD Biosciences) was used followed by donkey Alexa fluor 555 (Invitrogen)-conjugated anti-mouse secondary antibody. Mouse IgG (Santa Cruz) was used as isotype control. NOS2 staining was done with purified polyclonal rabbit anti-mouse NOS2 antibody (BD Biosciences) with rabbit IgG as isotype control, followed by Alexa fluor 647 (Invitrogen)-conjugated goat anti-rabbit secondary antibody. Samples were analyzed in a FACS Calibur flow cytometer (BD Immunocytometry Systems) and the data were analyzed using the FlowJo software (Tree Star).

### Labeling of conidia and Phagocytosis Assay

Live conidia were labeled with FITC (Sigma) according to previously described methods [71]. For the phagocytosis assay, alveolar macrophages were isolated from BAL and were cultured in complete RPMI media. Alveolar macrophages were incubated with FITC-labeled conidia for 4 hours at 37°C. At the end of the incubation period, phagocytosis was stopped by washing the macrophages with cold PBS and fixing cells with 4% PFA. Cells were collected and percent phagocytosis was analyzed by flow cytometry. To locate FITC-labeled conidia phagocytosed by macrophages, live cell imaging was done using a Nikon A1 Confocal on a Nikon Ti-E live cell microscope and data was analyzed with NIS-Elements imaging software. Cell tracker (Red CMPTX, Invitrogen) was used to stain cell cytoplasm and nuclei were stained with Hoechst.

### Statistical analyses

All statistical analyses were carried out using Graph Pad Prism software (Version 4). Student's unpaired two-tailed t-test was used for all statistical analyses. Differences between groups were considered significant when P<0.05.

## Supporting Information

Figure S1 Kinetics of gene expression in the lungs of Aspergillus-infected mice. (A) Mice were infected with various doses of RC or given PBS intratracheally and lungs were harvested after 48 hours of infection for total RNA extraction. Quantitative RT-PCR was performed to measure mRNA expression corresponding to various AAM markers. (B) Mice were infected with 50×10^6^ RC or given PBS intratracheally and lungs were harvested after various time points for total RNA extraction. Quantitative RT-PCR was performed to measure mRNA expression corresponding to various AAM-expressed genes. The fold increase was calculated relative to gene expression from PBS treated group after normalization to Gus-β (data are mean±SEM).
(TIF)
