# Supplementary figures and images for: Rapid Host Defense against Aspergillus fumigatus Involves Alveolar Macrophages with a Predominance of Alternatively Activated Phenotype
Source: PLoS One. 2011 Jan 5;6(1):e15943. doi: 10.1371/journal.pone.0015943 (PMC3016416; doi:10.1371/journal.pone.0015943)

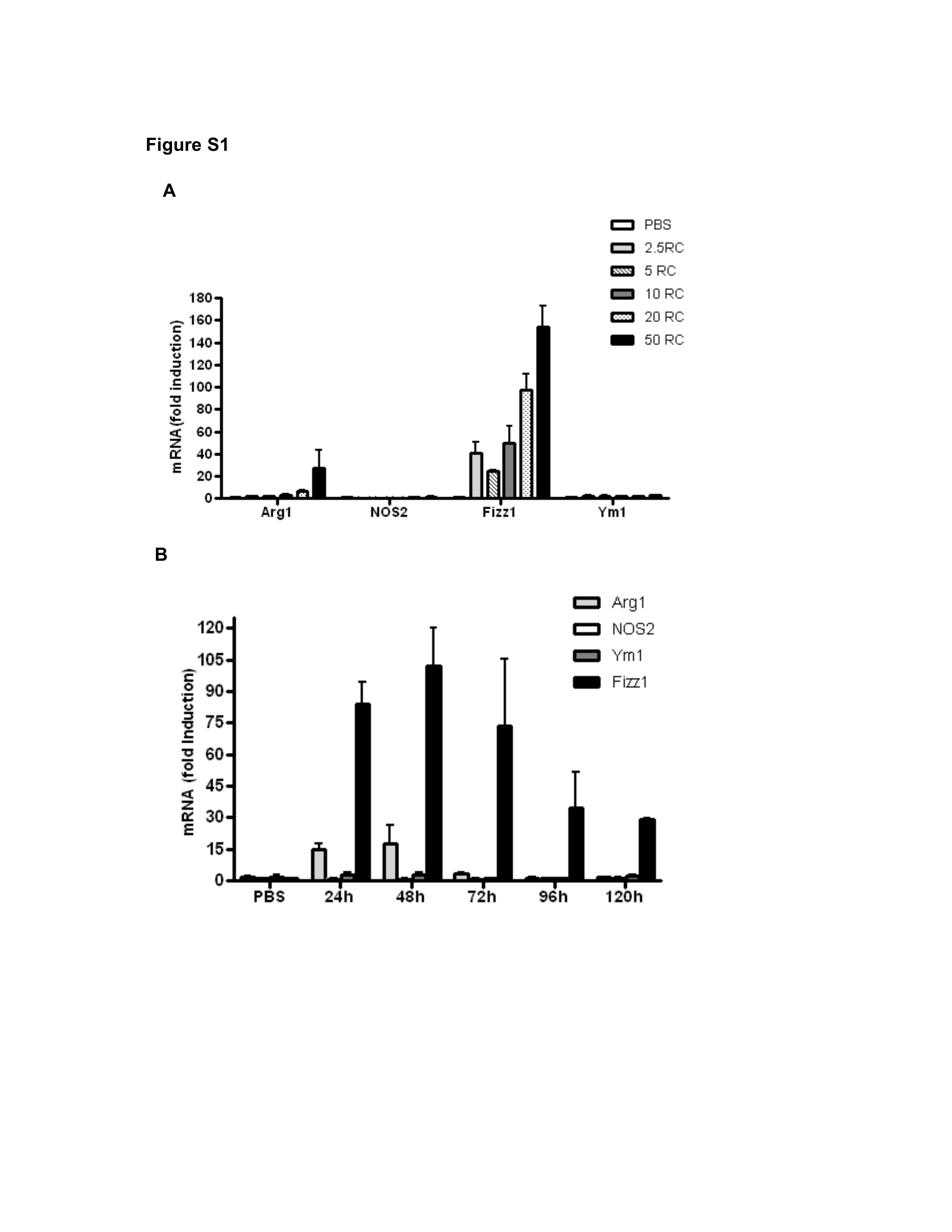

Supplement: Figure S1 — Kinetics of gene expression in the lungs of Aspergillus-infected mice. (A) Mice were infected with various doses of RC or given PBS intratracheally and lungs were harvested after 48 hours of infection for total RNA extraction. Quantitative RT-PCR was performed to measure mRNA expression corresponding to various AAM markers. (B) Mice were infected with 50×106 RC or given PBS intratracheally and lungs were harvested after various time points for total RNA extraction. Quantitative RT-PCR was performed to measure mRNA expression corresponding to various AAM-expressed genes. The fold increase was calculated relative to gene expression from PBS treated group after normalization to Gus-β (data are mean±SEM). (TIF) [file pone.0015943.s001.tif]
